# Effects of Varying Nitrogen Concentrations on the Locule Number in Tomato Fruit

**DOI:** 10.3390/plants14060952

**Published:** 2025-03-18

**Authors:** Meihua Sun, Jing Li, Linlin Tian, Huixian Sun, Yanxiu Miao, Longqiang Bai, Leiping Hou, Tianlai Li

**Affiliations:** 1 College of Horticulture, Shanxi Agricultural University, Jinzhong 030801, China; i12020020@163.com (J.L.); 13224759541@163.com (L.T.); 18686199372@163.com (H.S.); miaoyanxiu@163.com (Y.M.); lqbai@sxau.edu.cn (L.B.); sxndhlp@126.com (L.H.); 2 College of Horticulture, Shenyang Agricultural University, Shenyang 110866, China; tianlaili@126.com

**Keywords:** tomato, nitrogen, flower bud differentiation, cytokinin, fruit locule

## Abstract

Tomato seedlings were treated with nutrient solutions containing varying nitrogen concentrations (50, 150, and 250 mg·L^−1^) after germination until the completion of flower bud differentiation. The changes in nutrient content, enzyme activity, endogenous hormone levels, and gene expression in the stem apex were analyzed to explore the mechanisms regulating the number of locules in tomatoes at different nitrogen concentrations. The results indicated that an increase in nitrogen concentration facilitated the differentiation of tomato flower buds, increased the number of fruit locules, and increased the contents of soluble sugar, soluble protein, starch, and sucrose, as well as the activities of the enzymes POD, NR, and PPO in the seedling stem apex. The contents of soluble sugars and soluble proteins, as well as the activities of POD, NR, and PPO, were closely correlated with the number of fruit locules. An increase in nitrogen concentration was also found to elevate cytokinin levels while reducing auxin content in the stem apex. The transcriptome analysis screened for peroxidase genes, auxin response genes, and cytokinin synthesis genes. The analysis of gene expression patterns suggests that *CKX* and *LOG6* play significant roles in flower development. Additionally, combined physiological changes indicated that an increase in nitrogen concentration during the tomato seedling stage leads to a higher number of fruit locules, which may be associated with elevated cytokinin content, primarily involving the key genes *CKX* and *LOG6*.

## 1. Introduction

The tomato (*Solanum lycopersicum* L.) is an important vegetable crop in protected cultivation. However, the occurrence of malformed fruits in such environments can severely impact both the quality of tomatoes and their economic viability. An abnormal increase in the number of locules within tomato fruits can lead to malformation, potentially linked to factors such as irregular gene expression, exogenous application of plant hormones, and low temperatures.

Irregular gene expression can lead to an increased number of fruit locules and the occurrence of malformed fruit. Studies have demonstrated that the inverted mutation of the *fasciated* locus in tomatoes disrupts the regulatory region of the *CLAVATA3* gene, resulting in the downregulation of *CLAVATA3* gene expression, an increased number of locules, and a higher incidence of malformed fruit [1,2,3]. Two SNP mutations in the tomato *LOCULE NUMBER* (*lc*) locus altered the regulatory elements of the candidate gene *WUSCHEL*, resulting in increased expression and, consequently, a higher locule number of fruit [3,4]. Additional genes associated with the increase in the locule number of fruit due to gene mutations include *FAB*, *FIN*, *BES1.8*, *TPL3*, *IMA*, *ENO*, among others [2,5,6,7,8].

Exogenous plant hormones, as well as changes in endogenous plant hormones, can influence the locule number of fruit. The exogenous application of GA_3_ and GA_4_ to tomato seedlings significantly increased the locule number in tomato fruits, whereas PAC decreased the locule number in tomato fruits [6,9,10]. Additionally, the exogenous application of IAA to tomato seedlings significantly reduced the locule number, while N-1-naphthylphthalamic acid increased the locule number in tomato fruits [6]. Silencing the gibberellin receptor gene *GID1* in cucumbers resulted in an increase in the number of carpels and locules in 50% of the fruits [11]. Tomato plants with mutants of the auxin response factor *ARF8a* and *ARF8b*, as well as those with *GH3.4* overexpression of the auxin synthase gene, all exhibited an increased number of fruit locules [12]. Furthermore, an increase in cytokinin content in tomatoes due to *Moniliophthora perniciosa* infection also led to an increase in the locule number of fruit [13]. Correlation analysis indicated that the content of GA, IAA, and ABA in the flower buds of tomato inbred aligns with multi-locule, and few-locule, as well as their progeny was consistent with the locule number of the fruit [14].

Low temperatures during the tomato seedling stage can slow down flower bud differentiation, resulting in an increased number of flower organs and locules in the fruit, ultimately leading to the production of malformed flowers and fruits [15,16]. This abnormal fruit development may be attributed to low temperatures affecting the levels of gibberellins (GA) and abscisic acid (ABA) in the flower meristem. Such conditions can induce the accumulation of callose, which subsequently disrupts the distribution of the stem cell maintenance factor WUS in the flower meristem, thereby affecting the WUS-CLV feedback system [15]. Additionally, the malformed fruit caused by low temperatures may be linked to a reduction in the expression of *GA2ox* genes and an accumulation of GA_1_ and GA_4_ in the flower buds [16].

In this study, we found that increasing nitrogen levels during the seedling stage significantly increased the number of locules in tomato fruit. By analyzing changes in nutrient content, enzyme activity, plant hormone levels, and gene expression in the tomato stem apex, we further investigated the mechanisms by which increased nitrogen nutrition increases the number of fruit locules.

## 2. Results

### 2.1. Effects of Varying Nitrogen Concentration on Tomato Flower Development

**Figure 1:**
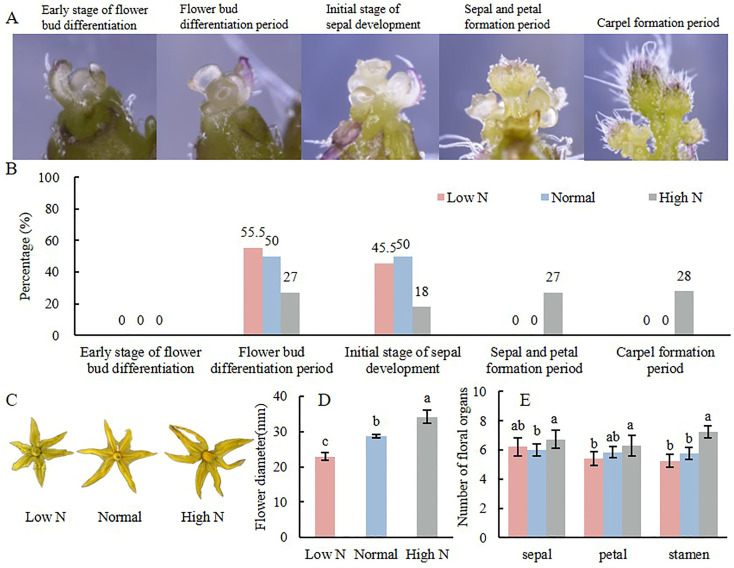
Effects of varying nitrogen concentrations on tomato flowers bud differentiation process (**A**,**B**), flower diameter (**C**,**D**), and number of floral organs (**E**). Note: Different lowercase letters indicate significant differences between treatments (*p* < 0.05).

The process of tomato flower bud differentiation was observed under a stereoscopic microscope and was categorized into five stages: the early stage of flower bud differentiation, the flower bud differentiation stage, the initial stage of sepal development, the sepal and petal formation period, and the carpel formation period (Figure 1A). Statistical analysis of the tomato flower bud differentiation process at varying nitrogen concentrations revealed that higher nitrogen levels accelerated the flower bud differentiation process. Among the flower buds treated with low N, 50% were in the flower bud differentiation stage, while the other 50% were in the initial stage of sepal development. In contrast, 55.5% of the flower buds treated with normal nitrogen were in the flower bud differentiation stage and 44.5% were in the initial stage of sepal development. For the high N treatment, 27% were in the flower bud differentiation stage, 18% were in the initial stage of sepal development, 27% were in the sepal and petal formation period, and 28% were in the carpel formation period (Figure 1B).

The effects of varying nitrogen concentrations on tomato flowers were observed. The results indicated that as nitrogen concentration increased, the flowers became significantly larger (Figure 1C). Specifically, the flower diameter of high N was significantly increased by 19.3% compared to normal, whereas the diameter of low N decreased significantly by 20% compared to normal (Figure 1D). There was no significant change in the number of sepals and petals across the three treatments. However, the number of stamens was significantly higher in high N compared to both the normal and low N. Flowers of high N had 7.25 stamens, while those with normal and low N had 5.75 and 5.25 stamens, respectively, (Figure 1E).

### 2.2. Effects of Varying Nitrogen Concentration on Tomato Fruit

**Figure 2:**
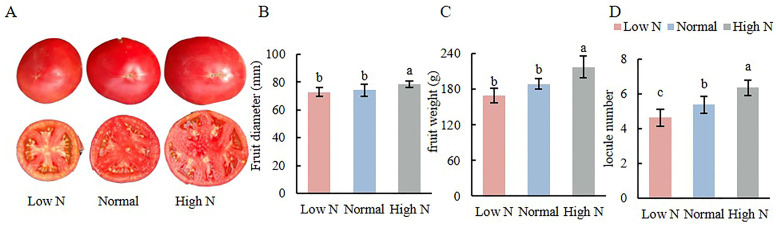
Effects of varying nitrogen concentrations on tomato fruit (**A**), fruit diameter (**B**), fruit weight (**C**), and locule number (**D**). Note: Different lowercase letters indicate significant differences between treatments (*p* < 0.05).

The effects of varying nitrogen concentrations on tomato fruits were analyzed, revealing that fruits treated with high N exhibited significantly larger sizes and a greater number of locules compared to those treated with low N and normal N (Figure 2A). Specifically, under high N conditions, fruit diameter and weight increased significantly by 5.3% and 15.3%, respectively, compared with normal conditions; however, no significant differences were observed between the normal and low N conditions (Figure 2B,C). When comparing high N conditions to normal conditions, the fruit diameter and weight increased significantly by 5.3% and 15.3%, respectively, while fruit diameter and weight under low N conditions decreased significantly by 12.81% and 24.5%. Furthermore, the fruits of high N conditions had an average of six locules, which was significantly higher than the five locules found in normal fruits and the four locules in low N fruits (Figure 2D).

### 2.3. Effects of Varying Nitrogen Concentration on Nutrient Content and Enzyme Activity in the Stem Apex of Tomato Seedlings

**Figure 3:**
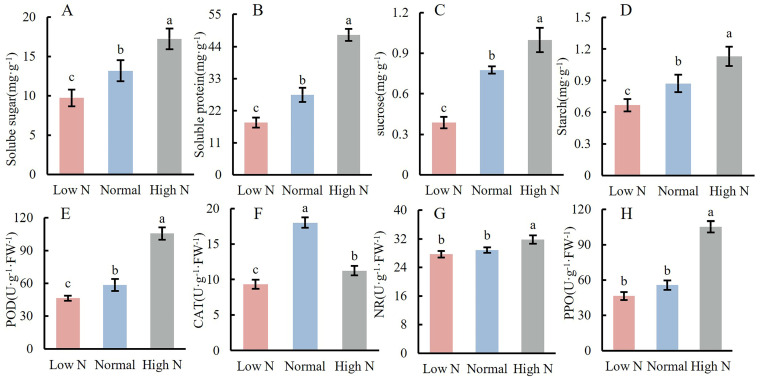
Effects of varying nitrogen concentrations on nutrient content and enzyme activity in the stem apex of tomato seedlings. Note: Different lowercase letters indicate significant differences between treatments (*p* < 0.05).

The analysis of nutrient content changes in the stem apex of tomato seedlings revealed that the contents of soluble sugar and soluble protein in high N were significantly increased by 30.8% and 75.2%, respectively, compared to normal. In contrast, the low N conditions resulted in a significant reduction of 26.4% and 34.6% for soluble sugar and soluble protein, respectively, relative to the normal conditions (Figure 3A,B). Furthermore, the contents of sucrose and starch in high N conditions were significantly increased by 28.9% and 29.4%, respectively, compared to normal conditions, while the low N conditions led to significant decreases of 50.2% and 23.7%, respectively, compared to the normal conditions (Figure 3C,D).

The changes in the enzyme activity in the stem apex of the tomato seedlings were analyzed, revealing that the POD (peroxidase) activity of the high N condition was significantly increased by 80.5% compared to normal conditions. In contrast, the POD activity of the low N condition was significantly decreased by 20.6% compared to normal conditions (Figure 3E). The CAT (catalase) activity of the high N and low N conditions was significantly decreased by 37.7% and 48.2%, respectively, compared to normal conditions. Notably, CAT activity of high N was significantly increased by 20.3% compared to low N (Figure 3F). Furthermore, the activities of NR (nitrate reductase) and PPO (polyphenol oxidase) under high N conditions were significantly increased by 10.1% and 89.2%, respectively, compared to normal conditions, while no significant differences were observed between normal and low N conditions (Figure 3G,H).

Correlation analysis showed that the contents of soluble protein and soluble sugar, as well as the activities of POD and PPO, were closely associated with flower diameter, fruit diameter, weight, and locule number. In contrast, the contents of starch, sucrose, and the activities of NR and CAT had little effect on these parameters (Table 1).

### 2.4. Effects of Varying Nitrogen Concentration on Hormone Content in the Stem Apex of Tomato Seedlings

**Figure 4:**
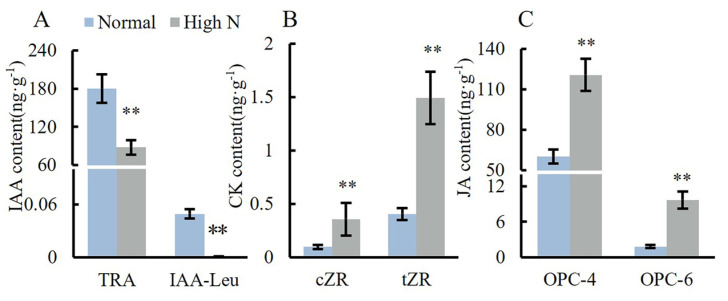
Effects of varying nitrogen concentrations on hormone content in the stem apex of tomato seedlings. Note: ** means significant at *p* < 0.01 level.

The contents of IAA-Leu and the precursor TRA in high N conditions were significantly reduced by 98% and 51.1%, respectively, compared to normal conditions (Figure 4A). Conversely, the contents of the cytokinin cZR and tZR in high N conditions were both significantly increased, showing an increase of 2.7 times compared to normal conditions (Figure 4B). Additionally, the contents of the Jasmonic acid precursors OPC-4 and OPC-6 in high N conditions were significantly increased by 1 time and 4.5 times, respectively, compared to normal (Figure 4C).

### 2.5. Transcriptome Data Analysis

Analysis of the stem apex transcriptome in normal and high N-treated seedlings revealed the detection of 269 differentially expressed genes. Among these, 162 genes were upregulated, while 107 genes were downregulated (Figure S1A). GO database enrichment analysis indicated that the differentially expressed genes were significantly enriched in response to heat, secondary metabolite biosynthetic processes, secondary metabolic processes, and flavonoid biosynthetic processes, among others (Figure S1B). KEGG analysis showed that these genes were mainly enriched in flavonoid biosynthesis, flavone and flavonol biosynthesis, protein processing in endoplasmic reticulum, anthocyanin biosynthesis, etc. (Figure S1C).

### 2.6. Effects of Varying Nitrogen Concentration on Expression of Related Genes in the Stem Apex of Tomato Seedlings

Genes related to soluble sugar, soluble protein, POD, PPO, auxin, and cytokinin synthesis, as well as locule number regulation, were selected from the differentially expressed genes identified in the transcriptome. The expression changes in these genes between normal and high N conditions were verified, demonstrating that the expression changes in the selected genes align with the transcriptomic data.

**Figure 5:**
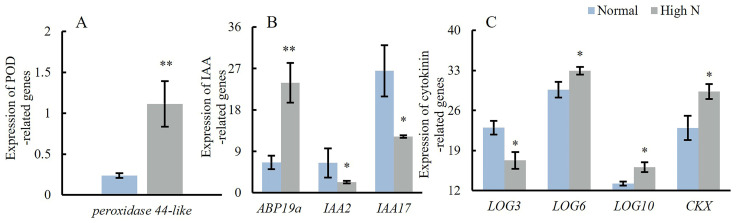
Effects of varying nitrogen concentrations on gene expression in the stem apex of tomato seedlings. Note: * means significant at *p* < 0.05 level, ** means significant at *p* < 0.01 level.

The *PRX44-like* (*peroxidase 44-like*) gene, which encodes a peroxidase, was significantly upregulated in high N conditions (Figure 5A). The *ABP19a*, responsible for encoding the auxin-binding protein ABP19a, was also significantly upregulated in high N conditions. Conversely, the *IAA2* and *IAA17* genes, which encode auxin-responsive proteins, were significantly downregulated in high N conditions (Figure 5B). *LOG3*, *LOG6*, and *LOG10* genes encoded cytokinin riboside 5′-monophosphate phosphoribohydrolase; *LOG3* was significantly downregulated in high N conditions, whereas *LOG6* and *LOG10* were significantly upregulated under the same conditions. Additionally, *CKX*, which encodes cytokinin oxidase/dehydrogenase-like, was significantly upregulated in high N conditions (Figure 5C).

### 2.7. Analysis of Expression Patterns of Candidate Genes in Different Tissues

**Figure 6:**
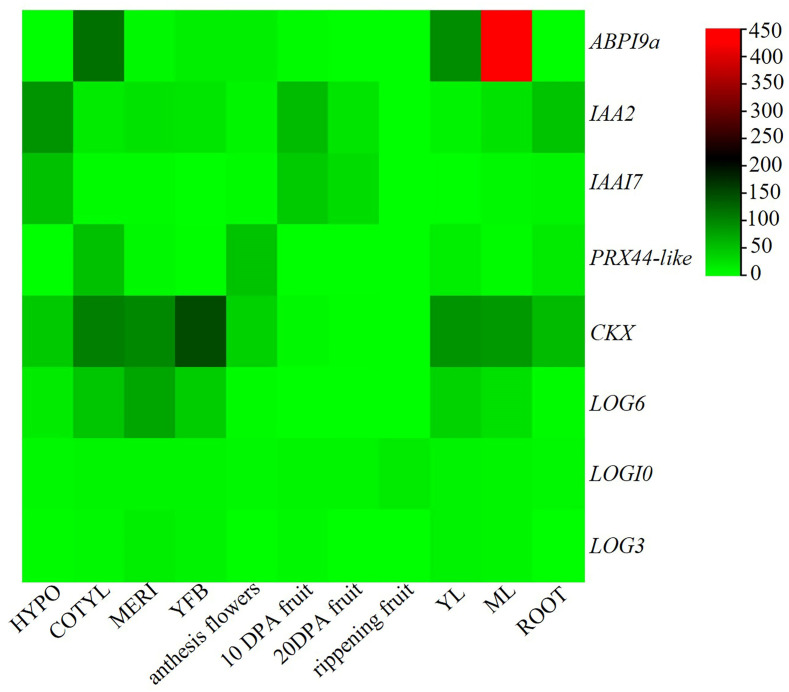
Expression heatmap of candidate genes in various tissues. Note: The legend represents the normalized RPKM value, red indicates a high expression level, and green indicates a low expression level.

Heatmaps depicting gene expression across various tissues indicated that nine candidate genes were expressed in a total of 11 tissues. Notably, the expression of *ABP9a* was higher in cotyledons, young leaves, and mature leaves. In contrast, *IAA2* and *IAA17* exhibited higher expression in hypocotyls, as well as 10 days post anthesis fruits and 20 days post anthesis fruit and roots. Additionally, *PRX44-like* demonstrated significant expression in cotyledons and anthesis flowers. *CKX* and *LOG6* were found to be more highly expressed in cotyledons, meristems, young flower buds, young leaves, and mature leaves. *LOG10* and *LOG3* exhibited comparable expressions across all 11 tissues (Figure 6).

## 3. Discussion

### 3.1. Effects of Varying Nitrogen Concentration on Tomato Flower Development and Fruit

Numerous studies have indicated that enhancing nitrogen nutrition during the seedling stage can facilitate flower bud differentiation. An increase in nitrogen concentration prior to and during the period of flower bud differentiation not only promotes this differentiation but also leads to larger flower sizes [17]. Conversely, early nitrogen deficiency in strawberry seedlings has been shown to inhibit flower bud differentiation, while an appropriate nitrogen concentration fosters this process [18,19]. Research on cereal crops has consistently demonstrated that a moderate increase in nitrogen nutrition at the seedling stage enhances the transition from vegetative to reproductive growth [20]. This study further corroborates that augmenting nitrogen supply during the seedling stage can promote flower bud differentiation in tomatoes. Additionally, our findings reveal that increased nitrogen supply correlates with larger flower sizes and a higher number of stamens.

It is widely recognized that an increase in nitrogen supply correlates with enhanced crop yields; however, the impact of elevated nitrogen concentration during the seedling stage on yield remains underexplored. This study demonstrates that augmenting nitrogen nutrition during the seedling phase significantly increases both the size and weight of tomato fruits, suggesting a potential rise in overall tomato yield. Additionally, the findings indicate that higher nitrogen supply at the seedling stage leads to an increase in the number of locules in tomato fruits. Moreover, temperature is a critical factor influencing the number of carpels and locules in tomatoes. The study was conducted in a greenhouse equipped with temperature control, maintaining a relatively stable environment at 26 °C/18 °C.

However, the temperature environment in tomato-protected cultivation is unstable, leading to differing effects of increased nitrogen concentration on the number of carpels and locules in tomatoes compared to this study. While an appropriate increase in nitrogen fertilizer can enhance yield, excessive nitrogen application can negatively impact crop quality. Specifically, applying too much nitrogen does not further increase yield; instead, it decreases the sugar-to-acid ratio in the fruit, resulting in diminished fruit quality. Additionally, excessive nitrogen fertilizer has been shown to reduce the solids-to-acids ratio and anthocyanin content in blackberry fruit [21].

### 3.2. Effects of Varying Nitrogen Concentration on Nutrient Content, Enzyme Activity and Hormone Content in the Stem Apex of Tomato Seedlings

The contents of soluble sugar, soluble protein, sucrose, and starch in the stem apex, as well as the activities of POD, CAT, NR, and PPO, increased with rising nitrogen concentration during the seedling stage. Correlation analysis indicated that the levels of soluble sugar, soluble protein, and the activities of POD and PPO were closely associated with the number of fruit locules. Carbohydrates serve as a crucial material basis for flower bud differentiation in plants [22]. Research on apples (*Malus pumila* Mill.) has demonstrated that a high carbohydrate content is advantageous for flower bud formation [23]. Furthermore, soluble sugar is one of the factors regulating the flowering of cherry (*Prunus pseudocerasus*) [24], and a high soluble sugar content facilitates the flower bud differentiation of cherries [25]. In this study, it was demonstrated that an increase in nitrogen concentration during the seedling stage led to a rise in soluble sugar content in the stem apex, subsequently accelerating flower bud differentiation.

Antioxidant oxidases can effectively remove ROS from plants, which have been implicated in the growth of various floral organs during early development [26]. Furthermore, elevated ROS concentrations in the leaves of *Litchi chinensis* were found to promote flower bud differentiation and flowering [27]. This study also showed that, under high nitrogen treatment, the activity of POD and PPO in the stem apex increased leading to accelerated flower bud differentiation. Furthermore, it suggests that an appropriate increase in nitrogen fertilizer can enhance the antioxidant system of plants, thereby improving their adaptability to adverse conditions [28]. Additionally, as nitrogen concentration increased during the seedling stage, the number of locules in tomato fruit also increased, while the content of auxin in the stem apex decreased and cytokinin content increased. This observation aligns with previous studies indicating that the exogenous application of auxin inhibitors, coupled with an endogenous rise in cytokinin, increases the number of locules in tomato fruits [6,13].

### 3.3. Effects of Varying Nitrogen Concentration on Gene Expression in the Stem Apex of Tomato Seedlings

The transcriptome analysis identified the peroxidase gene *PRX44-like*, auxin response genes *ABP19a*, *IAA2*, and *IAA17*, as well as cytokinin synthesis genes *LOG6*, *LOG10*, and *CKX*. The expression patterns of these candidate genes indicated that *CKX* and *LOG6* genes are highly expressed in meristem and young flower buds, suggesting their significant roles in flower development.

In summary, the increased nitrogen concentration during the tomato seedling stage leads to an increase in the number of fruit locules, potentially linked to elevated cytokinin levels, specifically involving the key genes *CKX* and *LOG6*. The Arabidopsis (*Arabidopsis thaliana*) *CKX* gene family comprises seven members, which are involved in regulating leaf area size, apical meristem size, flowering time, and root growth [29]. In contrast, the rice *CKX* gene family consists of eleven members, with functions that are diversified in regulating rice (*Oryza sativa* L.) growth and development, particularly in the control of tillering [30]. The Arabidopsis *LOG* gene family consists of eight members and plays a crucial role in regulating the size of the apical meristem, as well as stem and root growth [31]. In contrast, the rice *LOG* gene family comprises ten members, with primary functions that include the regulation of apical meristem size, floral organ number, and pistil development [32]. Although there are limited studies on the *CKX* and *LOG* gene families in tomatoes, it is speculated—based on the identification of gene functions in other species—that *CKX* and *LOG* genes are significant in regulating apical meristem size and flower development.

Nitrogen serves not only as a vital nutrient for plant growth and development but also functions as a signaling molecule that regulates gene expression. For example, NLP (NIN-like protein) acts as a transcription factor that senses nitrate signals. The Arabidopsis NLP8 protein binds to the promoter of the *CYP707A2* gene which encodes abscisic acid decomposase, thereby enhancing its expression; this represents the molecular mechanism by which nitrate promotes seed germination in Arabidopsis thaliana [33]. Additionally, maize (*Zea mays* L.) NLP3.2 negatively regulates the expression of *Aux/IAA14* through its interaction with the ARF19 protein, illustrating the molecular mechanism by which low nitrogen regulates root biomass [34]. Consequently, the regulation of *CKX* and *LOG6* expression by nitrogen in this study may also involve NLP proteins. Future research will aim to further elucidate the roles of key NLP transcription factors. In future studies, knockout mutants of *CKX* and *LOG6*, as well as overexpressed plants, will be constructed. Bioinformatics analyses and molecular biology experiments will be employed to further investigate the molecular mechanisms regulating nitrogen concentration and the number of locules in tomato fruit.

## 4. Materials and Methods

### 4.1. Plant Materials

The tomato ‘Zhongshu4’ was selected as the plant material for this study. Fully developed seeds were selected, and, after germination, they were sown in a medium composed of sand, perlite, and vermiculite in a 1:1:1 ratio. The seedlings were cultured in a greenhouse maintained at a day/night temperature of 26 °C/18 °C. Following seedling emergence, nutrient solutions with low nitrogen (N) as well as normal and high N concentrations were applied separately. The formula for the nutrient solutions is presented in Table 2. These solutions were applied every two days until the completion of flower bud differentiation in the seedlings that had developed three true leaves. The flower and inflorescence meristems of the seedlings were frozen in liquid nitrogen and stored at −80 °C for the analysis of physiological indices, transcriptomes, and gene expressions. Additionally, 20 seedlings from each treatment were selected to observe flower bud differentiation, while 30 seedlings were planted in a solar greenhouse to investigate flower and fruit morphology.

### 4.2. Flower and Fruit Phenotypic Analysis

The Stereo Discovery V8 microscope (ZEISS, Baden-Württemberg, Germany) was utilized to observe the flower bud differentiation stage with 20 plants examined per treatment. Ten flowers at the anthesis stage were selected from three treatments, the number of sepals, petals, and stamens in the flowers was recorded, and the diameter of the flowers was measured using a ruler. Ten mature fruits from three treatments were collected, and the number of fruit locules was assessed; the weight of the fruit was measured using a balance, and the diameter of the fruit was also measured with a ruler.

### 4.3. Physiological Index Analysis

The content of soluble protein was analyzed using the Coomassie Brilliant Blue G-250 staining method [35]. The contents of soluble sugar and starch were determined by the anthrone colorimetric method [36] while sucrose content was analyzed via spectrophotometry [37]. The results were expressed as mg·g^−1^ (fresh weight). The methods for detecting POD and PPO activity were based on the protocols established by Lee et al. [38]. The activities of CAT and NR were assessed using the methods described by Grajeda-Iglesias and Poonnachit [39,40]. The results were expressed as U·g^−1^·FW^−1^ (fresh weight).

The sample was ground to powder using liquid nitrogen and subsequently extracted with 1 mL of a methanol/water/formic acid solution in a volume ratio of 15:4:1. After concentration, 100 μL methanol solution (80%) was added to the sample bottle. The samples were then analyzed using Ultra Performance Liquid Chromatography (UPLC) on the ExionLC^™^ AD system (https://sciex.com.cn/) (SCIEX, Foster, CA, USA) and Tandem Mass Spectrometry (MS/MS) with the QTRAP^®^ 6500+ (https://sciex.com.cn/) (SCIEX, Foster, CA, USA). For details regarding the liquid phase conditions, refer to Cai et al. [41], and for mass spectrum conditions, consult Pan et al. [42].

### 4.4. Transcriptome Sequencing and Bioinformatics Analysis

Total RNA (5 μg) was extracted from the flower and inflorescence meristems of seedlings using a plant RNA extraction kit (CWBiotech, Beijing, China). The concentration and purity of the RNA were assessed using a NanoDrop 2000 (Thermo Fisher Scientific, Waltham, MA, USA), and RNA integrity was verified through 1% agarose gel electrophoresis. Transcriptome sequencing was performed by Shanghai Meiji Biomedicine Technology Co., LTD. (Shanghai, China), with the sequencing library constructed using Illumina^®^ Stranded mRNA and Ligation (Illumina, San Diego, CA, USA), followed by sequencing on the NovaSeq X Plus platform (Illumina, San Diego, CA, USA). The criteria for screening differentially expressed genes were set at *p* < 0.05 and log2FC ≥ 1. Gene ontology analysis of the differentially expressed genes was conducted using the Gene Ontology database (http://www.geneontology.org/), and KEGG pathways analysis of the differentially expressed genes was analyzed based on the KEGG pathways database (http://www.genome.jp/kegg/, accessed on 9 January 2025).

### 4.5. RNA Extraction and Related Gene Expression Analysis

The RNA was extracted from the flower and inflorescence meristems of seedlings using a plant RNA extraction kit DP452 (Tiangenbio, Beijing, China). The quality of the RNA was assessed via agarose gel electrophoresis, while its purity and concentration were measured using an enzyme label (Tecan Infinite M200, Tecan, Männedorf, Switzerland). cDNA was synthesized using a reverse transcription kit KR118 (Tiangenbio, Beijing, China). Gene expression analysis was conducted, and the primers utilized are listed in Table 3. The TB Green fluorescence quantitative kit (Sangon Biotech, Shanghai, China)) on an ABI7500 system with ABI7500 software v2.3 was used for analysis. The *ACTIN* gene was used as the reference to calculate the relative gene expression.

The IBM SPSS Statistics 23.0 software package was utilized to analyze significant differences with * indicating significant differences at *p* < 0.05, ** indicating significant differences at *p* < 0.01 according to the Independent *t*-test. Small letters indicate significant differences at *p* < 0.05 according to Duncan’s multiple range tests. Origin 2021 software was used for drawing

### 4.6. Analysis of Gene Expression Patterns

RNA-Seq data were obtained from the Tomato Genome Database (https://solgenomics.net/, accessed on 23 January 2025) to analyze the expression patterns of candidate genes in tomato tissues. The Transcripts Per Million (TPM) values were utilized as the measure of gene expression, and a heat map was generated using TBtools-II software.

## 5. Conclusions

Malformed fruit is frequently produced in tomato-protected cultivation, with an abnormal increase in the number of fruit locules being a common cause of this malformation. This increase in fruit locules can result from low temperatures during the seedling stage, excessive application of plant hormones, and abnormal gene expression. Our study reveals that elevated nitrogen concentrations in tomato seedlings also led to an increase in the number of fruit locules. Further investigation into the underlying mechanisms revealed that as nitrogen concentration increased, there was a corresponding rise in the content of soluble sugar, soluble protein, sucrose, and starch, as well as the activities of POD, NR, and PPO in the stem apex during early flower development. Correlation analysis indicated that the contents of soluble sugar, soluble protein, the activities of POD, NR, and PPO were closely related to the number of fruit locules. Additionally, as nitrogen concentration increased during the seedling stage, the content of cytokinin in the stem apex rose, while auxin content decreased. Transcriptomic analysis identified genes associated with these factors, and the analysis of gene expression pattern highlighted the cytokinin-related genes *CKX* and *LOG6* as key players. In conclusion, the increase in nitrogen concentration during the tomato seedling stage contributes to the rise in the number of fruit locules, likely linked to the increased cytokinin content in the stem apex during early flower development, with *CKX* and *LOG6* identified as critical genes.

## Figures and Tables

**Table 1:** Correlation analysis of nutrient content and enzyme activity in the stem apex of seedlings with flower and fruit traits.

Item	Soluble Protein	Starch	Sucrose	Soluble Sugar	POD	NR	CAT	PPO
Flower size	0.94 **	0.911 **	0.981 **	0.898 **	0.934 **	0.834 **	0.211	0.879 **
Fruit size	0.947 **	0.937 **	0.903 **	0.971 **	0.897 **	0.802 **	0.202	0.903 **
Fruit weight	0.942 **	0.869 **	0.820 **	0.916 **	0.899 **	0.794 **	0.071	0.919 **
Locule number	0.850 **	0.747 *	0.708 *	0.872 **	0.856 **	0.721	0.180	0.811 **

Note: * means significant at *p* < 0.05 level, ** means significant at *p* < 0.01 level.

**Table 2:** Nutrient solution formulations for different treatments (large element) mg/L.

Treatment	KNO_3_	Ca(NO_3_)_2_·4H_2_O	NaH_2_PO_4_·4H_2_O	NH_4_NO_3_	CaCl_2_	MgSO_4_	Total Amount of Fertilizer		
							N	P_2_O_5_	K_2_O
Normal	301	912	395.5	--	--	400	150	180	140
High N	301	912	395.5	286	--	400	250	180	140
Low N	301	70	395.5	--	748	400	50	180	140

**Table 3:** Primers used in real-time fluorescence quantitative experiments.

Gene	Primer Sequence
ABP19a-F	CCCAAGTGGTGTCATCCCAT
ABP19a-R	AGGAATGTTGTTGCCGCAAC
IAA2-F	AAGTAGTTGGATGGCCACCG
IAA2-R	TACGTCACCAGCAAGCATCC
IAA17-F	TGCCTGGTGGAGCAAGAAAA
IAA17-R	TAAGGTGCACCATCCATGCT
PRX44-like-F	AAGTTCTGCAACGGCGTTTC
PRX44-like-R	TCTCCAGCACTCCCCACTAA
LOG3-F	GGAGGAGGTAGCATAGGCCT
LOG3-R	TACCGGCTTATCGTGGATGC
LOG6-F	AAGTGATAACCTGGGCGCAA
LOG6-R	CCAAGTTGTTCCGTCTCCCA
LOG10-F	ATACACAGGCTTGGTCGTGG
LOG10-R	CCTTGCGGAGAAAAACCTGC
CKX-F	AATCGATGGTCAGTGGCGTT
CKX-R	GCCAGGTGCAAGGACATACT
ACTIN-F	5′-GATCAGCGTATCCTTCAGAG-3′
ACTIN-R	5′-GGCATTGTAGCAGAGAAAAC-3′

## Data Availability

The original contributions presented in the study are included in the article; further inquiries can be directed to the corresponding author.

## Supplementary Materials

The following supporting information can be downloaded at https://www.mdpi.com/article/10.3390/plants14060952/s1, Figure S1: Transcriptome analysis of the stem apex in normal and high N treated seedlings.
