# Supplementary material for: Effects of Varying Nitrogen Concentrations on the Locule Number in Tomato Fruit
Source: Plants (Basel). 2025 Mar 18;14(6):952. doi: 10.3390/plants14060952 (PMC11944714; doi:10.3390/plants14060952)
Supplement: Supplementary file 1 [file plants-14-00952-s001.zip › plants-3484830-supplementary.pdf]

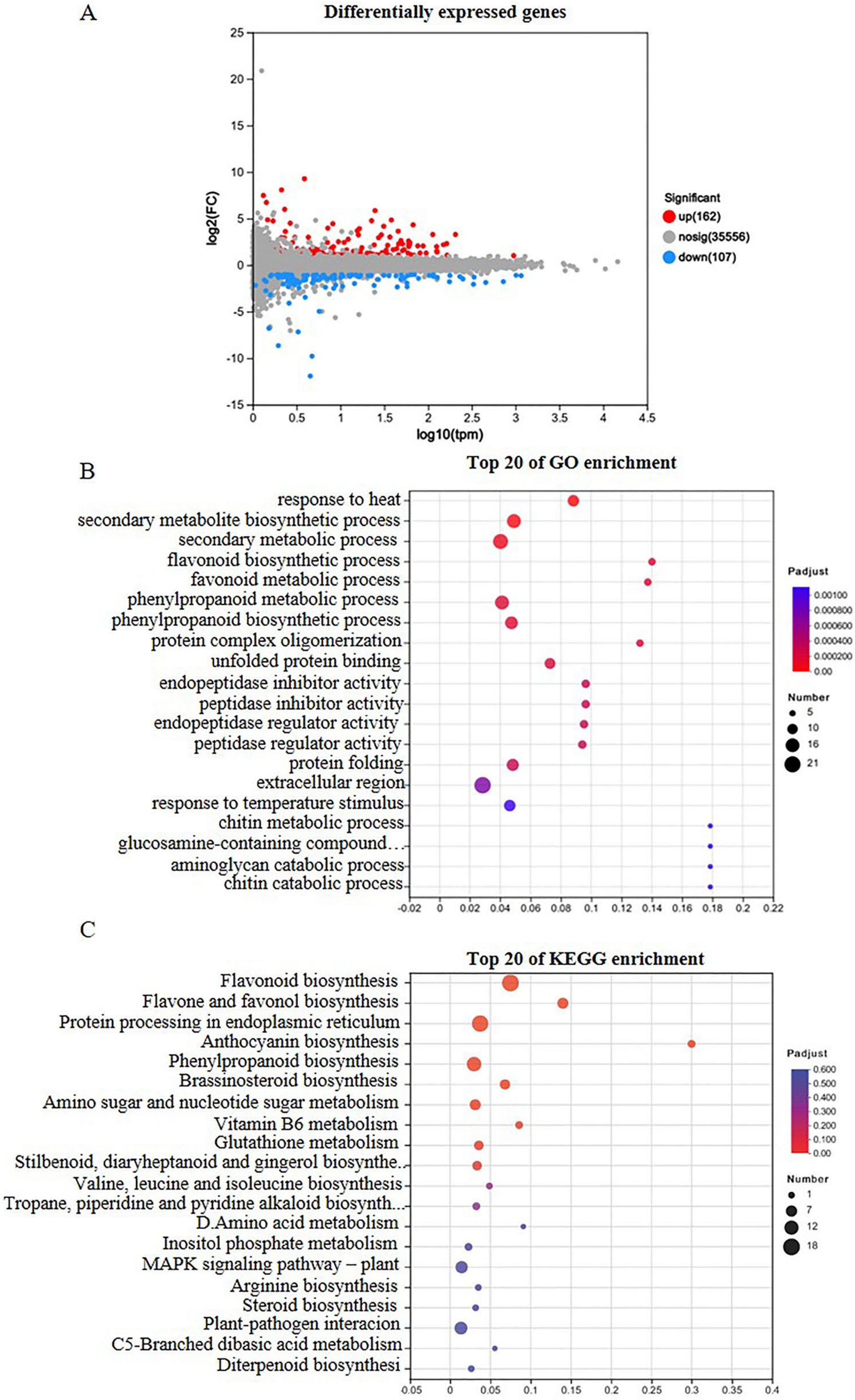

Figure S1. Transcriptome analysis of the stem apex in normal and high N treated seedlings: (A) Differentially expressed genes; (B) GO functional annotation of the differentially expressed genes. (C) KEGG enrichment analysis of the differentially expressed genes. Differentially expressed genes were selected based on their absolute fold change value of logFC > 1 and a p-value < 0.05. p-value < 0.05 was used as the standard for screening enriched GO data and KEGG pathway databases.
